# Periodontitis and implant complications in diabetes

**DOI:** 10.1111/prd.12451

**Published:** 2022-08-01

**Authors:** Luigi Nibali, Nikolaos Gkranias, Giuseppe Mainas, Antonino Di Pino

**Affiliations:** ^1^ Periodontology Unit Centre for Host Microbiome Interactions Faculty of Dentistry, Oral & Craniofacial Sciences King's College London London UK; ^2^ Centre for Immunobiology and Regenerative Medicine and Centre for Oral Clinical Research Institute of Dentistry Queen Mary University London (QMUL) London UK; ^3^ Department of Clinical and Experimental Medicine University of Catania Catania Italy

**Keywords:** diabetes, periodontitis, peri‐implantitis

## Abstract

Epidemiologic evidence indicates that periodontitis is more frequent in patients with uncontrolled diabetes mellitus than in healthy controls, suggesting that it could be considered the “sixth complication” of diabetes. Actually, diabetes mellitus and periodontitis are two extraordinarily prevalent chronic diseases that share a number of comorbidities all converging toward an increased risk of cardiovascular disease. Periodontal treatment has recently been shown to have the potential to improve the metabolic control of diabetes, although long‐term studies are lacking. Uncontrolled diabetes also seems to affect the response to periodontal treatment, as well as the risk to develop peri‐implant diseases. Mechanisms of associations between diabetes mellitus and periodontal disease include the release of advanced glycation end products as a result of hyperglycemia and a range of shared predisposing factors of genetic, microbial, and lifestyle nature. This review discusses the evidence for the risk of periodontal and peri‐implant disease in diabetic patients and the potential role of the dental professional in the diabetes‐periodontal interface.

## 1 BACKGROUND

Diabetes mellitus and periodontal disease are among the most common chronic diseases of mankind^1^, ^2^, ^3^ and, remarkably, they share many common features. Periodontal diseases encompass a group of diseases affecting the supporting apparatus of the teeth, including gingiva, root cementum, periodontal ligament, and alveolar bone. The most common forms of periodontal diseases, gingivitis and periodontitis, are characterized by a microbially driven series of host responses that lead to periodontal tissue damage.^4^, ^5^ The host response is central to the development of periodontitis, as it is to the development and progression of several human chronic diseases, including diabetes mellitus. In the next sections we will review the main features of diabetes mellitus, with the main focus being the association between type 2 diabetes mellitus and periodontitis.

### 1.1 Diabetes mellitus

Diabetes mellitus is a complex chronic disease requiring continuous and multiple interventions on glycemic targets and cardiovascular risk factors in order to prevent acute and chronic complications. According to the current World Health Organization classification, there are two major types of diabetes mellitus: type 1 and type 2 (https://www.who.int/publications/i/item/classification‐of‐diabetes‐mellitus). The two forms are heterogeneous diseases in which physiopathology, clinical presentation, and disease progression vary considerably.

Type 1 diabetes, previously called “insulin‐dependent diabetes,” accounts for 5%‐10% of diabetes and is due is due to autoimmune beta‐cell destruction, usually leading to absolute insulin deficiency. It is defined by the presence of one or more autoimmune markers, including islet cell autoantibodies and autoantibodies to glutamic acid decarboxylase (glutamic acid decarboxylase 65), insulin, tyrosine phosphatases IA‐2 and IA‐2b, and zinc transporter 8. The disease has strong human leukocyte antigen associations, with linkage to the *DQA* and *DQB* genes.^6^

Type 2 diabetes, previously referred as “non–insulin‐dependent diabetes” or “adult‐onset diabetes,” accounts for 90%‐95% of all diabetic cases. The core pathophysiologic defects in type 2 diabetes include beta‐cell failure and insulin resistance in muscle and liver.^7^ Although it is often associated with a strong genetic background, the genetic profile of type 2 diabetes is poorly understood, and various genetic and environmental factors can result in insulin resistance and progressive loss of beta‐cell mass and/or function that manifest clinically as hyperglycemia. According with this consideration, type 2 diabetes occurs more frequently in certain racial/ethnic subgroups (African American, American Indian, Hispanic/Latino, and Asian American).^8^ Furthermore, the risk of developing type 2 diabetes increases with age, obesity, and lack of physical activity.^9^ Indeed, obesity and decreased physical activity are strictly bounded to insulin resistance status and, when added to the genetic background, play a major role in the development of diabetic disease. In the preclinical stage of type 2 diabetes, pancreatic beta‐cells augment insulin secretion to offset the defect of insulin action. As long as the beta‐cells manage to increase insulin secretion the glucose plasma levels remain normal or near normal; but when beta‐cell function fails, the plasma glucose concentration starts to rise, leading to the onset of overt type 2 diabetes.^6^, ^10^, ^11^ Although loss of beta‐cells function and insulin resistance are the core defects of type 2 diabetes, the adipose tissue, gastrointestinal hormones, alpha‐cell, kidney, and brain all play important roles in the pathophysiology of glucose intolerance.^7^

According to the American Diabetes Association guidelines,^12^ type 2 diabetes may be diagnosed based on plasma glucose criteria (fasting plasma glucose and 2‐hour plasma glucose after a 75 g oral glucose tolerance test) or glycated hemoglobin criteria^13^ (see Table 1).

**TABLE 1:** Diagnostic criteria for diabetes and prediabetes based on American Diabetes Association standards of medical care in diabetes^13^

	Diabetes mellitus	Prediabetes
Fasting plasma glucose	≥126 mg/dL (7.0 mmol/L)	≥100 and <126 mg/dL (≥5.6 and <6.9 mmol/L) (impaired fasting glucose)
2‐h plasma glucose during oral glucose tolerance test	≥200 mg/dL (11.1 mmol/L)	≥140 and <200 mg/dL (≥7.8 and <11 mmol/L) (impaired glucose tolerance)
Glycated hemoglobin	≥6.5% (48 mmol/mol)	≥5.7% and <6.5% (≥39 and <47 mmol/mol)
Other	Classic hyperglycemic symptoms or hyperglycemic crisis and a random plasma glucose ≥200 mg/dL (11.1 mmol/L)	

Lifestyle factor is an overall first‐line therapy for preventing and managing type 2 diabetes.^14^ However, effective treatment of type 2 diabetes often requires several drugs used alone or in combination to correct the multiple physiopathological abnormalities of this disease.^15^ Metformin and pioglitazone correct insulin resistance in liver and muscle, respectively, and decrease the hepatic glucose production that is characteristic of type 2 diabetes.^16^, ^17^ Sulfonylureas increase plasma insulin level, stimulating its secretion from beta cells,^7^ and glucagon‐like peptide‐1 analogues and dipeptidyl peptidase IV inhibitors enhance, directly and indirectly, gastrointestinal hormones plasma levels (incretins), resulting in stimulation of insulin secretion.^18^ α‐Glucosidase inhibitors inhibit the breakdown of complex carbohydrates in the gastrointestinal tract, leading to delayed carbohydrate absorption and reduction in postprandial hyperglycemia.^19^ Finally, sodium‐glucose co‐transporter‐2 inhibitors (the newest class of oral agents) inhibit the renal glucose transporter, resulting in increased urinary glucose excretion.^20^

Insulin is the cornerstone of therapy for type 1 diabetes. However, many patients with type 2 diabetes will eventually require and benefit from insulin therapy. In fact, because of the progressive decline of beta‐cell function, blood glucose often becomes inadequately controlled with oral glucose‐lowering treatments or incretin‐based therapies only. At that stage, supplementary insulin therapy is typically added.^21^

The main problem in the management of individuals with type 2 diabetes is the high risk of development of micro‑ and macrovascular diseases, which have two distinct pathogenic sequences, leading to two distinct clinical presentations.^22^ Microvascular complications (retinopathy, nephropathy, and neuropathy) are a major cause of morbidity. However, cardiovascular disease (myocardial infarction, stroke, congestive heart failure, and peripheral artery disease) is the leading cause of mortality in patients with diabetes, accounting for 80% of all deaths.^23^, ^24^, ^25^ Although microvascular complications could be reduced through improved glycemic control from glucose‐lowering drugs, the importance of glucose control for reducing macrovascular disease has been highlighted only in recent clinical trials conducted with new classes of hypoglycemic drugs (sodium‐glucose co‐transporter‐2 inhibitors and glucagon‐like peptide‐1 agonists).^26^ It is clear that management of cardiovascular disease risk in type 2 diabetes requires management of multiple risk factors, and current treatment guidelines recommend the aggressive management of blood glucose and cardiovascular disease risk factors in these patients.^27^

Diabetes mellitus has reached epidemic status in the United States. To date, more than 32 million Americans are suffering from diabetes,^2^ with direct and indirect costs estimated reaching above $327 billion a year.^2^, ^28^ The high prevalence and the prognostic implications of diabetes mellitus has increased the interest on diabetes mellitus prevention programs. In particular, some of these prevention programs have aimed at identifying subjects at high risk of developing type 2 diabetes. According with these considerations, in 1997 and 2003, the Expert Committee on Diagnosis and Classification of Diabetes Mellitus identified a clinical condition characterized by a hyperglycaemia that does not meet the diagnostic criteria for diabetes mellitus and defined it as “prediabetes.”

### 1.2 Prediabetes

Prediabetes is a general term that refers to an intermediate stage between normal glucose homeostasis and overt type 2 diabetes mellitus. Similar to type 2 diabetes, prediabetes may be diagnosed based on plasma glucose criteria or glycated hemoglobin criteria^13^ (see Table 1).

Subjects with isolated impaired fasting glucose seem to have a reduced hepatic insulin sensitivity, impaired first‐phase insulin secretion, and normal/near‐normal muscle insulin sensitivity, whereas subjects with impaired glucose tolerance are characterized by nearly normal hepatic insulin sensitivity and marked reduced peripheral insulin sensitivity combined with defective late insulin secretion.^29^, ^30^ In contrast to impaired fasting glucose and impaired glucose tolerance, glycated hemoglobin is a marker representing blood glucose concentrations over the preceding 2‐3 months and it is affected by both basal and postprandial hyperglycemia. To date, it is still not clear if these aspects that are strictly bound to the physiopathology of prediabetes may have a clinical relevance in view of a possible therapeutic intervention.

In the absence of other comorbidities, prediabetes has often been considered as a “benign” condition at low risk for progression to type 2 diabetes and serious cardiovascular complications. However, data from observational studies indicate that prediabetic patients have a significantly higher risk of developing type 2 diabetes than the general population do: Subjects with a single incident of glycemic tolerance (impaired fasting glucose or impaired glucose tolerance) will progress to type 2 diabetes in 6% of the cases per year, which is significantly higher than normo‐glycemic subjects (0.5% per year). Type 2 diabetes progression occurs in 30%‐40% of cases in 3‐8 years, with 10% increase when there is more than one alteration of glycemic homeostasis (eg, impaired fasting glucose and impaired glucose tolerance together).^31^ In addition to the increased risk of developing type 2 diabetes, the incidence of cardiovascular disease and the associated mortality is significantly higher in subjects with prediabetes than those with normal glycemic tolerance.^32^ Prediabetes is often associated with obesity (especially abdominal or visceral), dyslipidemia characterized by high levels of triglycerides and low levels of high‐density lipoprotein cholesterol, and hypertension;^33^ moreover, prediabetes is a risk factor for a number of pathologies that are generally taken into consideration after patients have had a diagnosis of type 2 diabetes. Among them, we can include periodontal disease, cognitive decline, micro‑ and macrovascular pathology, low levels of testosterone, and hepatic steatosis.^34^, ^35^, ^36^, ^37^, ^38^, ^39^

In addition, the overwhelming majority of patients with prediabetes are unaware of their diagnosis, so it is crucial that they are identified, in particular in the presence of mild hyperglycemia, so that they can benefit from timely interventions on risk factors and lifestyle. Accordingly, the American Diabetes Association's recommendations point out to the importance of identifying prediabetic subjects for primary prevention interventions.^40^ To date, lifestyle modifications are the most important feature for preventing progression to type 2 diabetes and reducing the long‐term risk of cardiovascular disease.^9^ Whereas lifestyle interventions are recommended for all prediabetic patients identified by impaired fasting glucose, impaired glucose tolerance, and glycated hemoglobin, current guidelines recommend that pharmacological treatment (metformin) should be reserved for those patients with a double impairment of glucose tolerance (eg, impaired fasting glucose/impaired glucose tolerance) plus other risk factors such as hypertension, low high‐density lipoprotein cholesterol, high triglycerides, obesity, age <60 years or a family history of diabetes in a first‐degree relative.^41^ In these patients, strong consideration should be given to the management of cardiovascular risk factors with the following objectives:

- arterial hypertension <140/85 mmHg using angiotensin‐converting enzyme inhibitor or blocker of angiotensin receptor;
- low‐density lipoprotein cholesterol <100 mg/dL in prediabetic without history of cardiovascular disease;
- low‐density lipoprotein cholesterol <70 mg/dL in prediabetics with history of previous major cardiovascular events;
- high‐density lipoprotein cholesterol over 40 mg/dL in men and over 50 mg/dL in women;
- triglycerides <150 mg/dL;
- aspirin reserved for primary prevention for all high‐risk patients and for all secondary prevention patients;
- cessation of smoking habit.

### 1.3 Periodontal disease in diabetes: The “sixth” complication?

The association of diabetes mellitus and periodontal disease was proposed more than half a century ago,^42^ and since then this has been investigated and reported in numerous studies of diverse populations in different parts of the world. This volume of evidence has led to the proposed identification of periodontitis as the “6th complication of diabetes mellitus”.^43^

The studies that have investigated the diabetes mellitus‐periodontal disease association are mostly either population‐wide epidemiological studies or disease‐specific (either diabetes mellitus or periodontal disease) population cross‐sectional or longitudinal analyses. Overall, diabetes mellitus, in its two most common forms, type 1 diabetes and type 2 diabetes, has been associated with a higher prevalence of periodontal pathology than that of the general population.^44^, ^45^, ^46^, ^47^ Furthermore, gingival inflammation (gingivitis) has been reported to be significantly increased in poorly glycemically controlled populations of type 1 diabetes and type 2 diabetes.^48^, ^49^, ^50^, ^51^, ^52^, ^53^

Pivotal studies to the identification of this association were those performed in populations like the Pima Indians in Arizona, a group with a significant prevalence of type 2 diabetes.^54^ The original study, as well as its several follow‐up studies, showed that patients with diabetes mellitus had an almost three times higher chance (odds ratio 2.6; 95% confidence interval 1.0‐6.6) of developing periodontitis tha the rest of the population.^44^, ^54^, ^55^ Furthermore, longitudinal studies performed in populations of Native Americans and Alaskan Natives showed a significantly higher prevalence of severe periodontitis among diabetic than non‐diabetic patients (34% vs 19%).^56^ Overall in the adult US population, as described through the Third National Health and Nutrition Examination Study results, poorly controlled diabetes mellitus patients exhibited a much higher prevalence of severe periodontal disease than nondiabetic subjects did (odds ratio, 2.90; 95% confidence interval, 1.40‐6.03).^57^

Similar results have been observed in the rest of the world. In Europe, a study performed in Swedish adults (40‐70 years old) showed that diabetes mellitus patients were found to present increased amount of radiographic bone loss and probing pocket depth compared with nondiabetics.^47^ However, this difference reached significance only in the younger age group (40‑ to 49‐year‐olds) which led the authors to suggest early onset of diabetes mellitus as a higher risk factor than duration/exposure to it.^58^ A study in Finland^59^ indicated an association of the metabolic control rather than the presence of diabetes mellitus with the periodontal health status. In Italy, Campus et al,^60^ in an adult cross‐sectional study on 71 type 2 diabetes patients and 141 nondiabetic controls, found a significant association of diabetic status with periodontal health status as well as with plaque and bleeding indices. However, other studies, like a cross‐sectional study performed in Spain with 144 subjects, 70 of whom were diabetic (mixed type 1 diabetes and type 2 diabetes), only identified increased gingival index and loss of periodontal attachment in diabetes mellitus subjects but no differences in probing pocket depths.^61^

In a small (n = 23) South African type 2 diabetes group of patients, the prevalence of periodontal disease was significantly more prevalent in the “poorly controlled” ones (glycated hemoglobin >8.0%) when compared with the “well‐controlled” ones (glycated hemoglobin <8.0%) (42% versus 18%, *P* < 0.002).^62^ A recent systematic review concluded that people with periodontitis have a weighted higher mean glycated hemoglobin of 0.29% (95% confidence interval, 0.20%‐0.37%, *P* < 0.01) than periodontally healthy subjects do.^63^ Generally, wound healing in type 2 diabetes subjects seems to be impaired due to alterations in macrophage and cytokine responses, which reflect in suboptimal healing after nonsurgical and surgical periodontal procedures, as well as tooth extractions.^59^, ^64^, ^65^

Most previously mentioned studies included type 2 diabetes populations. However, there is also some evidence that young type 1 diabetes individuals, particularly those with poor metabolic control as assessed by glycated hemoglobin levels, present with poorer periodontal condition than healthy individuals.^66^, ^67^ Older type 1 diabetes patients seem to have considerably more periodontitis than healthy subjects or type 2 diabetes subjects, although this could partially be explained by their longer exposure to diabetic pathology.^68^, ^69^ Martins Chávarry et al,^70^ in a meta‐analysis of the then available cross‐sectional studies, concluded that there is significantly higher prevalence and severity of periodontitis in type 2 diabetes and (young) type 1 diabetes patients than in healthy controls.

In regard to long‐term periodontal health stability, it has been proposed that uncontrolled diabetes may affect the success of the periodontal treatment and the risk of periodontal disease progression and recurrence.^71^ In a long‐term study that assessed the presence of periodontitis and its treatment response in two groups of young adults, one presenting with type 1 diabetes and one healthy control group, it was found that type 1 diabetes patients with poor metabolic control also presented increased periodontitis recurrence as described by increasing probing pocket depths compared with the control group.^72^ Overall, a systematic review of 49 cross‐sectional and eight longitudinal studies of diabetes mellitus‐periodontal disease association, although indicating various methodological weaknesses in the available studies, concluded on a significant association of type 2 diabetes and periodontal disease with an increased clinical attachment level by 1.00 mm (95% confidence interval, 0.15‐1.84) and periodontal pocket depth by 0.46 mm (95% confidence interval, 0.01‐0.91) for diabetes mellitus patients compared with non–diabetes mellitus patients.^70^ Similarly, Taylor et al.^71^ reported an odds ratio of 4.2 for progression of periodontitis in patients with type 2 diabetes when compared with healthy controls. However, it is important to emphasize that there is evidence of successful periodontal treatment and long‐term periodontal health stability in some cohorts of patients with diabetes.^73^, ^74^, ^75^ These data combined together may support the notion that metabolic control in patients with diabetes mellitus plays an important role in periodontitis incidence and treatment response.^76^ An analysis of 4343 subjects from Third National Health and Nutrition Examination Study study gave an odds ratio of having periodontitis of 2.90 for diabetes mellitus patients with poor glycemic control (glycated hemoglobin 9% or greater) over just an odds ratio of 1.56 when the metabolic control was better (glycated hemoglobin 9% or less), compared with nondiabetic individuals.^57^

### 1.4 Clinical comorbidities of diabetes and periodontitis

As well as potentially sharing some similar risk factors, diabetes mellitus and periodontal disease patients have exceptionally similar tendencies to develop comorbidities that tend to cluster in the same individuals, and which are listed in Table 2. An array of epidemiologic and interventional studies has associated both diabetes mellitus and periodontal disease with a series of states and conditions ultimately culminating in elevated risks of developing cardiovascular disease. The conditions and the relative associated evidence are briefly discussed in the following.

**TABLE 2:** Diabetes and periodontitis comorbidities

	Diabetes	Periodontitis
Hypertension	Up to 75% of adults with diabetes also have hypertension^2^, ^79^	Periodontal disease is associated with a higher risk of hypertension^81^
Obesity	Obesity accounts for the most cases of diagnosed type 2 diabetes mellitus in adults^95^	Overweight, obesity, and weight gain are associated with periodontal disease^101^, ^102^, ^103^
Dyslipidaemia	Similar incidence of hypercholesterolemia as in the general population, but atherogenic lipid profile with increased number of small and dense low‐density lipoprotein particles, reduced high‐density lipoprotein concentration, and higher triglyceride levels^84^, ^85^	Increased low‐density lipoprotein and triglycerides, reduced high‐density lipoprotein; higher levels of small, dense low‐density lipoprotein^90^, ^91^, ^93^
Oxidative stress	Increased measures of oxidative stress in patients with type 2 diabetes^105^, ^106^	Periodontal disease is associated with an increased local and systemic oxidative stress and compromised antioxidant capacity^109^, ^110^
Systemic inflammation	Adipose and vascular tissue of insulin‐resistant patients are in a persistent condition of low‐grade inflammation and are infiltrated with several classes of immune cells^111^	Increased C‐reactive protein levels in periodontal disease compared with controls;^112^ reduced systemic inflammation following periodontal therapy^115^, ^116^
Arterial wall thickness	People with type 2 diabetes have a higher carotid intima media thickness compared with nondiabetic controls with an estimated difference of 0.13 mm after adjusting for traditional risk factors^26^	Increased carotid intima media thickness compared with controls^14^, ^123^
Endothelial dysfunction	Flow‐mediated dilation was found to be impaired in diabetic patients compared with nondiabetic individuals^125^, ^126^, ^127^	Worse flow‐mediated dilation of brachial artery in periodontal disease,^128^ with improvements after treatment^117^, ^129^
Arterial stiffness	Increased arterial stiffness in alterations of glucose homeostasis^34^, ^130^	Increased pulse‐wave velocity compared with health or gingivitis^134^, ^135^
Cardiovascular disease	Two‑ to fourfold excess risk of cardiovascular disease^83^, ^136^	Periodontal disease measures, including pocket depths, bleeding on probing, and number of teeth, associated with cardiovascular disease^137^, ^138^, ^139^
Cardiovascular death	80% of all deaths of diabetic patients are attributable to cardiovascular disease^23^, ^24^, ^25^	Increased risk of cardiovascular disease mortality^140^, ^141^, ^142^

*Note*: Where possible, systematic reviews and meta‐analyses are indicated as references.

#### 1.4.1 Arterial hypertension

Hypertension is a chronic condition characterized by elevated arterial blood pressure currently defined as values >140 mmHg systolic blood pressure and/or >90 mmHg diastolic blood pressure.^77^ Hypertension is linked with increased risk of cardiovascular events, estimated to double with each 20/10 mmHg incremental increase in systolic/diastolic blood pressure above 115/75 mmHg in 40‑ to 69‐year‐old individuals.^78^ Up to 75% of adults with diabetes are estimated to suffer from hypertension, and patients with hypertension alone often show evidence of alterations of glucose homeostasis/insulin resistance.^79^

A systematic review reported that periodontal disease is also associated with a higher risk of hypertension (odds ratio, 1.50; 95% confidence interval, 1.27‐1.78), with the limitations of lack of prospective follow‐up studies and studies heterogeneity.^80^ A more recent systematic review and meta‐analysis corroborated those findings. Authors concluded that patients with moderate to severe periodontitis have 20% increased risk of having hypertension compared with periodontally healthy patients and, vice‐versa, an increased prevalence of periodontitis was observed in patients with hypertension.^81^

#### 1.4.2 Dyslipidemia

Dyslipidemia indicates an abnormal amount of lipids, usually increased levels of cholesterol and triglycerides, in the blood circulation.^82^ Diabetic dyslipidemia plays a key role in cardiovascular diseases in patients with type 2 diabetes; moreover, type 2 diabetes is one of the most frequent causes of secondary dyslipidemia. Diabetic patients present an atherogenic lipid profile characterized by an increased number of small and dense low‐density lipoprotein particles, reduced high‐density lipoprotein concentration, and higher triglyceride levels.^83^, ^84^, ^85^ Insulin resistance plays a pivotal role in lipid abnormalities in these patients, as it induces increased lipolysis of adipose tissue with high plasma levels of free fatty acid and reduces apolipoprotein B100 degradation in the liver. Furthermore, it promotes the production and secretion of more atherogenic very low density lipoprotein from the liver with an increased plasma concentration of small dense low‐density lipoprotein and a reduction of high‐density lipoprotein particles. According with these considerations, multiple clinical trials have demonstrated the beneficial effect of pharmacologic lipid management in patients with diabetes with or without cardiovascular disease.^86^, ^87^, ^88^ Current guidelines recommend obtaining a lipid profile at the time of diabetes diagnosis. Furthermore, lifestyle modification and/or pharmacological therapy (if indicated) are highly recommended to improve lipid profile in all such cases.^15^, ^89^

Altered lipid profiles have long been reported in periodontitis patients.^48^ A systematic review and meta‐analysis revealed that chronic periodontitis patients present significantly higher serum levels of low‐density lipoprotein and triglycerides and lower high‐density lipoprotein than healthy subjects do, whereas no differences were found for total cholesterol levels.^90^ Furthermore, higher levels of atherogenic small, dense low‐density lipoprotein have been detected in periodontal disease patients.^91^, ^92^ In two recent cross‐sectional studies, the same group of authors found a positive relationship between periodontitis and the triglyceride/high density lipoprotein cholesterol ratio ≥2.3 (odds ratio 1.47) and a positive association between moderate and severe periodontitis and dyslipidemia, specifically 30% and 16% higher, respectively, than in periodontally healthy patients.^93^, ^94^

#### 1.4.3 Obesity

Obesity indicates the excessive accumulation of body fats, generally defined as >30 kg/m^2^. Obesity accounts for most cases of diagnosis with type 2 diabetes in adults,^95^ and excess body weight increases the risk of death from any cause and from cardiovascular disease in adults between 30 and 74 years of age.^96^ The increased incidence of diabetes in Western countries is strictly related to the epidemic obesity and physical inactivity.^97^ Obesity is an insulin‐resistant state (−29% insulin sensitivity); however, as long as beta‐cells produce a compensatory insulin secretion, glucose tolerance remains normal/near normal. When beta‐cell function starts to fail, plasma glucose levels begin to rise, leading to the onset of overt diabetes.^98^

Periodontitis patients have been found to have a higher body mass index and a higher incidence of obesity than periodontally healthy subjects do.^99^ Conversely, a systematic review and meta‐analysis demonstrated that a significantly higher level of gingival inflammation is observed in obese people than in nonobese people.^100^ Two separate systematic reviews have confirmed these associations and concluded that overweight, obesity, and weight gain are associated with periodontal disease, suspecting that increased amount of lipids could contribute to the propagation of the inflammatory response leading to severe periodontal disease.^101^, ^102^ Nevertheless, a 10‐year retrospective study showed that, in spite of an increased incidence of periodontal disease progression in obese subjects, obesity is not an independent risk factor for progression of periodontitis when confounders were analyzed simultaneously.^103^

#### 1.4.4 Oxidative stress

Hyperglycemia promotes the overproduction of reactive oxygen species, which activate several pathways strictly related with the development of diabetes complications: increased polyol pathway flux, increased advanced glycation end‐products formation, increased protein kinase C activation, and increased hexosamine pathway flux. It also directly inactivates two critical anti‐atherosclerotic enzymes: endothelial nitric oxide synthase and prostacyclin synthase. Furthermore, reactive oxygen species cause insulin resistance in peripheral tissues by affecting the insulin receptor transduction pathway, ultimately resulting in decreased expression of glucose transporter type 4 in the cellular membrane.^104^ Increased measures of oxidative stress have been detected in patients with type 2 diabetes.^105^, ^106^ Increased intracellular reactive oxygen species seem to be involved in vascular health of diabetic patients: they cause defective angiogenesis in response to ischemia, activate a number of proinflammatory pathways, and cause long‐lasting epigenetic changes that drive persistent expression of proinflammatory genes after glycemia is normalized (“hyperglycemic memory”).

Oxidative stress is also an important player in determining the “collateral damage” consisting of periodontal attachment and alveolar bone loss in response to subgingival bacteria. In particular, patients with periodontitis, especially early‐onset forms, have been found to have phagocyte abnormalities, including excessive superoxide production.^107^, ^108^ Evidence suggests that periodontal disease is associated with an increased local and systemic oxidative stress and compromised antioxidant capacity.^109^ Interestingly, a systematic review and meta‐analysis observed that oxidative stress biomarkers (total antioxidant capacity, malondialdehyde, nitric oxide, total oxidant status, 8‐hydroxy‐deoxyguanosine) increased in saliva and only malondialdehyde in gingival crevicular fluid of periodontitis patients compared with healthy patients.^110^ However, studies are still needed to clarify the extent of oxidative stress in periodontitis cases locally and its potential systemic impact.

#### 1.4.5 Systemic inflammation

In patients with insulin‐resistance states, low‐grade inflammation is considered as a major contributor toward the progression to overt type 2 diabetes and cardiovascular disease. The adipose and vascular tissue of insulin‐resistant patients are in a persistent condition of low‐grade inflammation; these tissues are infiltrated with several classes of immune cells, including monocytes, macrophages, and lymphocytes, resulting in secretion of adipokines and proinflammatory cytokines, including interleukin‐1β, tumor necrosis factor alpha, interleukin‐17, and interleukin‐6.^111^

Patients with periodontitis seem to have increased systemic inflammation, measured as increased leukocytes, and particularly neutrophils,^112^ and increased plasma C‐reactive protein compared with controls.^113^, ^114^ Periodontal treatment has been shown to reduce systemic inflammation in periodontal disease patients,^115^, ^116^ despite an initial acute‐phase response following periodontal therapy.^117^ Interestingly, a systematic review assessing the effect of periodontal therapy on serum levels of inflammatory markers in people with type 2 diabetes mellitus concluded that periodontal therapy reduces serum levels of tumor necrosis factor alpha and C‐reactive protein in type 2 diabetes individuals.^58^ Another systematic review reported that periodontal therapy contributes to the reduction of interleukin‐6 serum levels in patients with type 2 diabetes.^118^

#### 1.4.6 Arterial wall thickness

Thickening of arterial walls is a common finding of cardiovascular disease–induced atherosclerosis. Measurement of carotid intima media thickness is one method of calculating plaque burden; it is linked to an increased risk of cardiovascular events and can be used as a noninvasive marker of subclinical atherosclerotic disease.^119^ A meta‐analysis of 21 studies reported that people with type 2 diabetes have a higher carotid intima media thickness than nondiabetic controls do, with an estimated difference of 0.13 mm after adjusting for traditional risk factors.^26^ This difference corresponds to a 10‐year increase in age compared with age‐matched controls and is associated with nearly 40% increase of cardiovascular risk. Furthermore, patients with impaired glucose tolerance had 0.04 mm thicker carotid intima media thickness than controls did (statistically significant). These data are in line with previous findings and highlight that the atherosclerotic process begins even before glycemic values have reached diagnostic thresholds for a diagnosis of diabetes.^120^

Cross‐sectional data on 6017 persons taking part in the Atherosclerosis Risk in Communities study showed that severe periodontitis was associated with increased carotid intima‐medial thickness, after adjusting for confounders, providing an indication that periodontitis may play a role in cardiovascular disease.^14^ Several other studies have suggested that patients affected by periodontitis have increased carotid intima‐media thickness,^121^, ^122^ and this has been confirmed by a meta‐analysis.^123^

#### 1.4.7 Endothelial dysfunction

Endothelial dysfunction comprises multiple functional alterations in the vascular endothelium (impaired regulation of vasodilation and vasoconstriction, increased inflammatory activation), all of which are associated with cardiovascular disease. In type 1 diabetes, endothelial dysfunction is triggered by the hyperglycemia‐related metabolic alterations. The relationship between endothelial dysfunction and type 2 diabetes is more complex, and different factors may be involved; however, insulin resistance seems to be a central mechanism linking endothelial dysfunction and alterations of glucose homeostasis. In fact, normal insulin signaling in the vascular endothelial cells is anti‐atherogenic. In insulin resistance states such as obesity, the insulin receptor‐AKT1 pathway in endothelial cells is downregulated; consequently, endothelial nitric oxide synthesis and release are impaired. This endothelial dysfunction might contribute to increased risk of atherosclerosis in patients with insulin resistance.^124^

The flow‐mediated dilation method, carried out noninvasively with ultrasonography on the brachial artery, is a frequently used method for the assessment of endothelial dysfunction and as a surrogate measure of cardiovascular disease. Flow‐mediated dilation was found to be impaired in diabetic patients compared with nondiabetic individuals.^125^, ^126^, ^127^ Similarly, flow‐mediated dilation of the brachial artery was found impaired in periodontitis cases^128^ and was found to improve following periodontal therapy.^117^ A meta‐analysis demonstrated that periodontal disease diagnosis was associated with a mean difference in flow‐mediated dilation of 5.1% compared with controls and that a mean improvement of 6.6% between test and control was observed after periodontal treatment.^123^ A recent experimental study showed that induced periodontitis in mice led to endothelial dysfunction.^129^

#### 1.4.8 Arterial stiffness

Arterial stiffness is also a result of a process of atherosclerosis due to inflammation and also to accumulation of advanced glycation end‐products. Arterial stiffness can be measured by carotid‐femoral and carotid‐radial pulse‐wave velocity.

A large body of evidence supports the concept of increased arterial stiffness in alterations of glucose homeostasis.^34^, ^130^ These data are of clinical relevance because an increase in aortic stiffness, measured by aortic pulse wave velocity, is an independent predictor of mortality in diabetic patients.^131^ To explain the association between hyperglycemia and vascular complications in diabetes, multiple risk factors seem to be involved (such as hypertension and smoking). However, several studies have emphasized the role of advanced glycation end‐products, high reactive molecules that form spontaneously in the condition of chronic hyperglycemia. Increased levels of advanced glycation end‐products determine decreased turnover of collagen and elastin in large arteries. Elastic fibers undergo lysis and disorganization subsequent to their replacement by collagen and other matrix components. These events cause a loss of elasticity and induce stiffening.^132^

Chronic hyperglycemia and hyperinsulinemia also increase local activity of the renin‐angiotensin‐aldosterone system and the expression of the angiotensin type I receptor in vascular tissue, promoting the development of arterial stiffness and fibrosis.^133^

The inflammatory status of severe periodontitis, by mechanisms yet not completely understood, is also associated with arterial stiffness and increased pulse‐wave velocity was detected in a recent systematic review comparing patients with periodontitis with subjects with periodontal health or gingivitis.^134^ An interesting recent systematic review and meta‐analysis concluded that subjects with severe periodontitis presented significantly higher carotid‐femoral, carotid‐radial and brachial‐ankle pulse‐wave velocity values compared with either periodontally healthy/gingivitis subjects or mild periodontitis subjects.^135^

#### 1.4.9 Cardiovascular events/death

All the factors above contribute to the two‑ to fourfold excess risk of cardiovascular disease in patients with type 2 diabetes.^83^, ^136^ A striking 80% of all deaths of diabetic patients is attributable to cardiovascular disease.^23^, ^24^, ^25^ Measures of periodontal disease, including pocket depths, bleeding on probing, and number of teeth, have been associated with cardiovascular diseases, such as coronary heart disease and stroke.^137^, ^138^, ^139^ A few studies have reported an increased risk of cardiovascular disease mortality in periodontal disease patients.^140^, ^141^ However, a recent exhaustive systematic review and meta‐analysis pointed out that periodontitis and its ultimate sequela, edentulism, are associated with increased risk ratio of mortality due to cardiovascular disease (relative risk 1.47 and 2.03, respectively) and coronary heart disease (relative risk 2.58 and 2.98, respectively).^142^

Interestingly, the risk of being affected by nephropathy macro‐albuminuria and end‐stage renal disease is increased in patients with diabetes mellitus and periodontitis compared with those only with diabetes mellitus.^143^ A prospective study in 628 Pima Indians affected by type 2 diabetes showed that cardiorenal mortality (including ischemic heart disease and diabetic nephropathy) was increased approximately threefold in patients with severe periodontitis compared with subjects with no to moderate periodontitis.^144^

### 1.5 Periodontal disease and diabetes: Two aspects of the same metabolic disorder?

**FIGURE 1:**
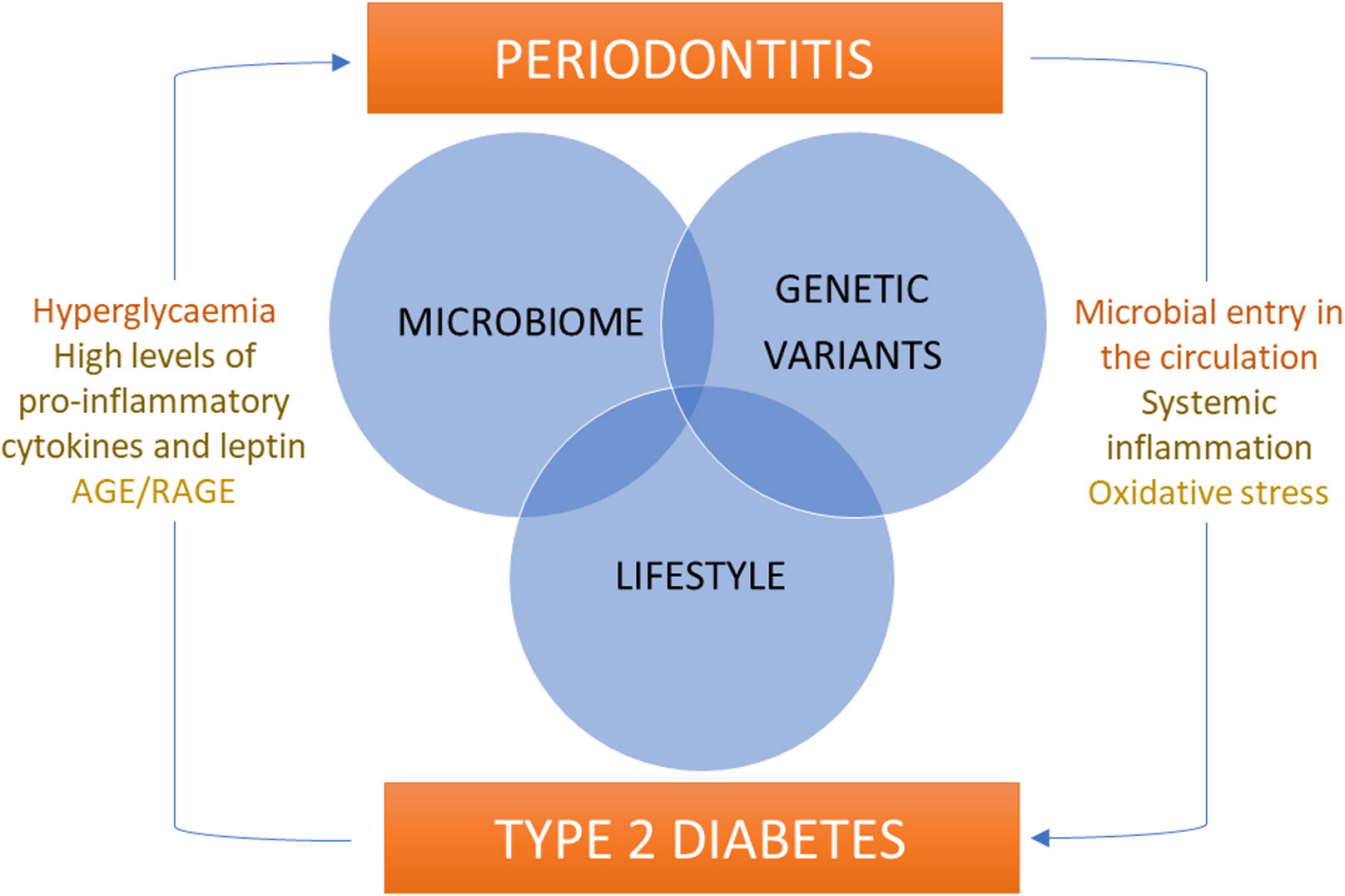
Schematic representation of relationships between type 2 diabetes and periodontitis. A combination of host genetic variants, microbiome, and lifestyle factors (some of them shared) seem to predispose to both conditions. Furthermore, a bidirectional association exists, with the presence of diabetes affecting periodontal disease and, in turn, periodontal disease affecting diabetes. AGE: advanced glycation end‐product; RAGE: receptor for advanced glycation end‐product

Glucose intolerance/insulin resistance, hypertension, obesity, dyslipidemia, arterial thickness, arterial stiffness, and cardiovascular events: Is it a coincidence that these aspects are common to both diabetes mellitus and periodontitis? Are both diseases' different aspects resulting from the same genetic‐microbial‐lifestyle–driven metabolic disorder? This theory is exemplified in Figure 1 and discussed in detail in the following.

#### 1.5.1 Genetics

Even though type 2 diabetes may be prevented with a healthy lifestyle and physical activity, some individuals appear more susceptible to the disease than others. Accordingly, evidence from twin and family studies has suggested a genetic basis of type 2 diabetes.^145^ A relatively small percentage (5% or less) of nonautoimmune diabetes is actually due to monogenic causes and is classified as monogenic diabetes of the young, whereas most other forms are probably “polygenic” in nature.^146^ The genetic architecture of type 2 diabetes diabetes might affect different mechanisms in its physiopathology. Dimas et al^147^ explored the relationship between type 2 diabetes genetic risk variants and indices of proinsulin processing, insulin secretion, and insulin sensitivity. They identified four variants associated with a clear insulin resistance pattern, two associated with reduced insulin secretion with normal fasting glycemia, and one with insulin processing. However, the variants identified in these studies collectively explain only a small portion of observed type 2 diabetes heritability (20%), and the missing heritability could be accounted by lower frequency variants and environmental determinants.^148^ Genome‐wide association studies identified a number of different loci with modest effect size associated with susceptibility to type 2 diabetes.^149^ A recent meta‐analysis indicated that carrying risk alleles in type 2 diabetes–associated genetic variants was associated with a modest risk of type 2 diabetes.^150^ Genetic background might also affect individual response to pharmacologic or lifestyle therapy. Accordingly, Pearson et al^151^ reported that transcription factor 7‐like 2 (*TCFL2*) genotypes are associated with a modest effect in the response to sulfonylurea treatment. To date, it is not clear whether genetic data might support the management of individual patients.

A recent systematic review estimated that up to a third of the variance of periodontitis risk in the population (heritability) is attributable to genetic variants.^152^ A polygenic multifactorial etiopathogenesis with several possible genes involved is the most likely for periodontal disease. Common single nucleotide polymorphisms affecting the host response to periodontal bacteria or affecting structural factors of the periodontium have been candidate as predisposing to periodontitis. A systematic review points to single nucleotide polymorphisms in the Vitamin D Receptor (*VDR*), the *Fc‐γRIIA*, and the Interleukin‐10 (*IL10*) as having the highest level of evidence for a role in periodontitis so far,^153^ although many more genes are likely to be involved in different populations. Recently, a systematic review and meta‐analysis found that tumor necrosis factor alpha rs1800629 polymorphism might increase the risk of developing periodontitis and diabetes concerning all genetic models, mainly in Asian subjects.^154^

#### 1.5.2 Microbiome

The oral cavity is heavily colonized by a relatively stable microbiota, second only to the colon for number of microbes in the human body. Oral microbes include bacteria, as well as viruses, archaea, fungi, and protozoa; they are organized in communities of bacteria termed biofilms in every surface of the oral cavity and are responsible for maintaining the oral health/disease balance.^155^ Changes in cell metabolism and growth within biofilms are determined by microbes that reside within a self‐produced matrix that protects the organisms from the host immune response. Periodontitis is a microbially driven disease, where the host response triggered by bacteria is thought to determine the formation of periodontal pockets, associated with periodontal attachment and bone loss.^4^ Periodontal pockets may in turn act as a microbial reservoir, which could determine the spread of bacteria into the bloodstream and potentially to other tissues and organs. In periodontitis, the ulceration of the pocket epithelium results in bacteremia even during chewing and tooth brushing.^156^ Although the survival of these bacteria in the systemic circulation and their final destiny are unclear, they have been hypothesized to have the ability to cause long‐ranging consequences. For example, deoxyribonucleic acid from periodontal bacteria has been detected in carotid atheromas by polymerase chain reaction and in the amniotic fluid.^157^, ^158^ Furthermore, keystone periodontal pathogen *Porphyromonas gingivalis* expresses peptidyl arginine deiminase, able to catalyze the enzymatic deamination of arginine to citrulline residues (in bacterial and host proteins) and thus to change the protein antigenic properties.^159^, ^160^ This citrullination provides a molecular mechanism for generating antigens that may break immune tolerance to citrullinated proteins, leading to an increased formation of autoantibodies, precipitating the host reaction leading to rheumatoid disease.^161^, ^162^ Recent experiments suggest that oral dysbiosis may even have an effect on gut microbial composition. In animals fed a high‐fat diet, *P. gingivalis* administration via cervical vein resulted in increases in triglycerides, body and liver weight and lipid accumulation, compared with sham‐administered mice, whereas administration of *Streptococcus sanguinis* and *Streptococcus salivalrius* had no such effect.^163^ A similar effect was noted following oral administration of *P. gingivalis* strain W83 twice a week for 5 weeks in mice, resulting in changes in the gut microbiota, increased blood endotoxin levels, insulin and glucose intolerance, and a decrease in gene expression of tight‐junction proteins in the ileum.^164^ In a different high fat–fed mouse model, insulin resistance was enhanced by pathogen‐induced periodontitis.^165^ Periodontopathogenic bacteria may also have systemic effects by production of short‐chain fatty acids, especially butyrate.^166^

Studies tend to point towards a dysbiotic microbiota in diabetic patients. The microbiota of type 2 diabetic subjects has lower species diversity and a lower abundance of butyrate‐producing bacteria (such as *Faecalibacterium prausnitzii*) and of some *Clostridium* clusters but a higher abundance of carbohydrate‐utilizing bacteria (such as lactic acid bacteria and bifidobacterial), possibly favored by a high‐sugar diet.^167^, ^168^ Germ‐free mice have been shown to be protected from diet‐induced obesity.^169^ A separate study showed that changes in gut microbiota have an influence on metabolic endotoxemia and inflammation, by increasing intestinal permeability, therefore suggesting a role for the gut microbiota in inducing obesity and potentially also insulin resistance.^31^

A rate of physiological bacterial translocation is thought to occur in the human gut, by the intra‐epithelial route and then via the mesenteric lymph nodes (or directly to the portal circulation in case of damage to the epithelium).^170^, ^171^ If the gut microbiota can indeed induce insulin resistance and obesity,^169^, ^172^ we could theorize an indirect role of the gut microbiota in increasing the risk to develop periodontitis. Or, if periodontal microbes such as *P. gingivalis* could actually affect the gut microbiota,^164^ this relationship could indeed be bidirectional. Furthermore, it is possible that one of the body's dysbioses might alter the biodiversity of the microbiota, provoking a loss of immunological tolerance to commensal bacteria, hence predisposing to other diseases in the body.^173^, ^174^ However, at this stage, it is still unclear whether the presence of diabetes affects the subgingival microbiota. A review commissioned by the European Federation of Periodontology and the American Academy of Periodontology concluded that neither diabetes (type I or II) nor glycemic control in diabetics have any clear effects on the composition of the periodontal microbiota.^175^ However, more recently, possible differences in microbial composition in diabetes mellitus patients compared with non–diabetes mellitus individuals have emerged from studies using 16S rRNA sequencing^176^, ^177^ and polymerase chain reaction.^178^, ^179^, ^180^ Some studies suggest that subgingival microbial differences may only be evident in poorly controlled diabetic patients.^178^, ^180^

#### 1.5.3 Lifestyle

According to the current guidelines, lifestyle is the first‐line therapy for preventing or delaying the onset of type 2 diabetes. The Diabetes Prevention Program demonstrated that intensive lifestyle intervention reduces the incidence of type 2 diabetes by 58% over 3 years.^181^ Other studies investigating the effect of lifestyle intervention in type 2 diabetes prevention reported similar findings.^9^, ^182^

After diabetes diagnosis, health status and quality of life are key goals of diabetes self‐management education and support that should be measured and monitored as part of routine care. All the patients should receive individualized diet compatible with American Diabetes Association recommendations and containing 50%‐55% carbohydrate, 20% protein, and 25% fat. Furthermore, all patients with diabetes should decrease the amount of time spent in daily sedentary behavior; physical activity is recommended two or three times per week. Recently, the effect of primary care–led weight management through low caloric diet (test) was investigated in comparison with pharmacological management (control) in type 2 diabetes. Diabetes “remission” was achieved in 46% of participants in the test group and 4% of participants in the control group, and remission increased according to weight loss.^183^ This study stresses the importance of lifestyle and diet management in type 2 diabetes. Finally, smoking cessation is required in all patients. Several studies conducted on diabetic populations demonstrated that smokers have an increased risk of micro‑ and macrovascular complications.^184^ We are not aware of any rigorous studies demonstrating that e‐cigarettes are a healthier alternative to smoking or can facilitate smoking cessation.

Recent decades have produced increasing evidence for a role of lifestyle factors in the predisposition to periodontitis. Probably the strongest lifestyle factor associated with periodontitis is oral hygiene, as increased dental plaque levels are directly related with presence of periodontal disease^185^ and response to treatment.^186^ Along with the strong evidence for the effect of tobacco smoking in increasing the risk to develop periodontitis,^187^, ^188^, ^189^ other lifestyle‐related factors have also emerged in more recent years, including obesity, a diet rich in fermentable carbohydrates (driving oxidative stress and advanced glycation end‐products as discussed earlier) and micronutrient deficiencies (vitamin C, D, and B12).^190^ It has thus been suggested that functional foods or probiotics could be helpful in periodontal disease management.^190^ However, recent summary evidence ^191^ does not support the use of functional foods, probiotics, dietary counseling or physicial exercise as part of periodontal treatment. ^189^ Instead, it is clear that smoking cessation, oral hygiene instruction, and motivation and control of obesity need to be considered as cornerstones in the prevention and treatment of periodontal diseases, along with professional plaque removal.^192^, ^193^

### 1.6 Bidirectional association mechanisms

As well as sharing many similarities from a pathogenic standpoint, as discussed earlier, diabetes mellitus and periodontal disease may directly influence one another as well as the comorbidities described in the previous paragraph in a complex web of interactions. For example, the microbially driven inflammatory response in periodontitis can be influenced by insulin resistance, obesity, and dysmetabolic state. Mechanisms include obesity‐associated low levels of adiponectin and high levels of proinflammatory cytokines and leptin, which may increase periodontal inflammation,^194^ immune responses,^195^ and oxidative stress,^196^ and increased inflammatory profile mediated by an influence on lymphocyte numbers and subpopulations.^197^ The hyperinflammatory tissue response typical of periodontitis is also exacerbated by insulin resistance.^60^ Adipocytokines further increase production of reactive oxygen metabolites, thus increasing oxidative stress, which is in turn associated with reduced pancreatic beta‐cell function and induction of insulin resistance.^198^ Insulin production also increases adiposity; hence, mechanisms of association can overlap.

As already mentioned, advanced glycation end‐products are the result of elevated blood glucose levels and in turn activate expression of receptor for advanced glycation end‐products, which contributes to impaired periodontal tissue repair in the presence of subgingival microbial triggers.^65^ Therefore, the relationship between diabetes mellitus and periodontal disease has been proposed to be bidirectional.^199^ Emerging evidence indicates that people with severe periodontitis have an increased risk of developing type 2 diabetes.^200^, ^201^ Analysis of the National Health and Nutrition Examination Study data (2973 subjects) led to the identification of periodontitis as an independent risk factor for developing diabetes mellitus.^202^ Furthermore, it has been shown that periodontitis can lead to insulin resistance.^203^ A recent systematic review and meta‐analysis of the available literature on the prevalence of diabetes mellitus in people with a clinical diagnosis of periodontal disease examined 29 studies and indicated an odds ratio of 2.59 for periodontal disease patients to have diabetes mellitus.^204^ On the basis of these facts, it was hypothesized that treatment of periodontitis, by means of nonsurgical debridement, could improve the diabetes mellitus metabolic control, and so a series of clinical trials were set up to investigate the hypothesis. Four meta‐analyses of the available randomized controlled trials^205^, ^206^, ^207^, ^208^ have independently identified an improvement in glycated hemoglobin levels of about 0.4%‐0.66% following nonsurgical periodontal treatment in patients with type 2 diabetes and periodontitis. The consensus report of the Joint Workshop on Periodontitis and Systemic Diseases, of the European Federation of Periodontology and American Academy of Periodontology in 2012, confirmed that improvement in glycated hemoglobin following periodontal treatment is possible.^76^ However, a randomized controlled trial of over 500 patients that took place in the United States did not show any improvement in glycated hemoglobin levels following nonsurgical debridement,^209^ generating doubts on the strength of a diabetes mellitus‐periodontal disease bidirectional hypothesis. A recent systematic review update suggests that the magnitude of reduction of glycated hemoglobin following periodontal treatment in type 2 diabetes patients seems to have significant benefits on systemic health.^210^ A more recent 12‐month randomized controlled trial on 264 patients again showed reductions in glycated hemoglobin in those receiving periodontal treatment compared with controls receiving only supragingival scaling and polishing.^211^ The adjunctive use of antibiotics to subgingival debridement does not enhance glycated hemoglobin reduction in type 2 diabetes.^212^

Not enough evidence is available to suggest whether an effect of periodontal treatment on glycated hemoglobin reduction exists in people with type 1 diabetes.

A retrospective study based on a dataset by Taiwan National Health Insurance that included 3039 and 12 156 type 2 diabetes subjects having “advanced periodontal treatment” and “non‐advanced periodontal treatment,” respectively, was recently carried out by Peng et al.^213^ They showed that advanced periodontal treatment was associated with reduction in the incidence of myocardial infarction and heart failure but not of stroke, suggesting that advanced periodontal therapy lowers the rate of cardiovascular disease in type 2 diabetes.

### 1.7 Peri‐implant complications in diabetes

Peri‐implant diseases are infectious conditions affecting dental implants, ranging from peri‐implant mucositis, which is an inflammatory lesion of the peri‐implant mucosa, to peri‐implantitis, which also affects the supporting bone.^214^, ^215^ According to the “2017 World Workshop on the Classification of Periodontal and Peri‐implant Disease and Conditions,” peri‐implant mucositis is a reversible inflammatory lesion of the peri‐implant mucosa characterized by bleeding on gentle probing (<0.25 N) and/or suppuration with or without increased probing depth compared with previous examinations, and absence of bone loss beyond crestal bone level changes resulting from initial bone remodeling.^216^ Peri‐implantitis is defined by presence of bleeding on probing and/or suppuration, increasing probing depth compared with previous examinations, and presence of bone loss beyond crestal bone level changes resulting from initial bone remodeling.^217^ In the absence of previous examination data, presence of bleeding and/or suppuration on gentle probing, probing depths of 6 mm or more, and bone levels of at least 3 mm (apical of the most coronal portion of the intraosseous part of the implant) will be considered.^218^

Both peri‐implant mucositis and peri‐implantitis are often defined as the “gingivitis” and “periodontitis” of implants respectively. However, peri‐implantitis may differ from periodontitis in the inflammatory cells involved in the lesion and in the progression rate. Furthermore, mucositis lesions may progress to peri‐implantitis earlier than their counterparts around teeth.^219^ A systematic review and meta‐analysis estimated a prevalence of mucositis that ranged from 19% to 65% and peri‐implantitis that ranged from 1% to 47%, whereas the weighted mean prevalence for mucositis and peri‐implantitis was 43% and 22%, respectively.^220^ In addition, a case series study with a 21‐26 years follow‐up observed a prevalence of 54.7% of mucositis cases and 22.1% of peri‐implantitis cases.^221^ However, a different view on peri‐implant diseases also exists, which sees progressive bone loss threatening implant survival as a very rare event and questions the existence of “peri‐implantitis” as a disease entity, suggesting that the microbially driven inflammatory reaction is a late complication secondary to adaptive bone response to surgical trauma and implant loading. In these authors' view, marginal bone loss around implants is in the great majority of cases associated with immune‐osteolytic reactions (“foreign‐body reactions”).^222^, ^223^

Patients with previous periodontal disease have been shown to have an increased risk of peri‐implantitis compared with patients with no previous history of periodontitis.^224^, ^225^, ^226^ Along these lines, if we accept that peri‐implant diseases are inflammatory processes similar to periodontal diseases on teeth, it is easy to make the assumption that the presence of uncontrolled diabetes mellitus would increase the risk to develop peri‐implant diseases and/or implant failure or lack of osseointegration, for the reasons mentioned in the previous paragraphs. In agreement with this, a systematic review reported that poorly controlled diabetes negatively affects implant osseointegration both in rat models and in humans; however, in diabetic subjects with optimal serum glycemic control, osseointegration seems to occur successfully.^227^ In further support of this concept, a more recent review suggested that patients with poorly controlled diabetes (but not those with well‐controlled diabetes) suffer from impaired osseointegration, elevated risk of peri‐implantitis, and higher level of implant failure. The use of antibiotics and chlorhexidine might improve implant success.^228^ A recent meta‐analysis focused on “hyperglycemia” (defined as levels of glycated hemoglobin at least 5.7% or fasting plasma glucose of at least 100 mg/dL) as a potential risk factor for peri‐implantitis, revealing a 50% higher risk of peri‐implantitis in hyperglycemic vs normoglycemic subjects. The relative risk attributable to hyperglycemia seemed to increase among nonsmokers. However, no statistically significant association was detected between hyperglycemia and peri‐implant mucositis.^229^ A recent 12‐month follow‐up meta‐analysis reported that, despite being glycemic controlled, type 2 diabetes patients were associated with a higher risk of peri‐implantitis (marginal bone loss, bleeding on probing, and pocket depth were the parameters measured) compared with healthy patients.^230^ Other systematic reviews reported that type 2 diabetes patients are more prone to develop peri‐implant disease and bone loss; additionally, authors found that implant complications increased as glycated hemoglobin levels increased (hyperglycemia; eg, values of 8%), highlighting the importance of maintaining an adequate glycemic control.^231^, ^232^ A very recent systematic review pointed that poorly controlled diabetes mellitus patients have a higher rate of peri‐implantitis over time after implant placement and a lower survival rate in the long term than healthy patients do.^233^ Furthermore, authors observed that there was no difference in terms of survival rate when considering diabetes mellitus–controlled subjects and that implant success was improved when an adequate perioperative anti‐infective therapy, including administration of antibiotics and chlorhexidine, was used.^233^

However, some controversial reports also exist. A systematic review found that patients with type 2 diabetes presented a very high implant survival rate, ranging from 86.3% (24‐months follow‐up) to 100% (12‐months follow‐up).^234^ Interestingly, another systematic review and meta‐analysis did not find any difference in survival rate of immediately loaded implants between either well‐controlled or poorly controlled type 2 diabetes patients and nondiabetic patients.^235^ No differences were also found in marginal bone loss when conventional and immediate loading were compared.^235^

Overall, uncontrolled diabetes together with genetic predisposition, smoking, history of periodontitis, specific subgingival microbes, and residual subgingival cement are generally considered as predisposing to peri‐implant diseases.^226^, ^236^

### 1.8 Diabetes: The oral health professional's role

It remains to be confirmed whether type 2 diabetes and periodontitis are a manifestation of an overall metabolic/inflammatory disturbance. Nonetheless, there is no doubt that these two diseases are associated and tend to often occur in the same individuals. Therefore, given the also often‐delayed diagnosis of diabetes mellitus, the oral health professional (dentist, hygienist, therapist, nurse) could have an important role in prompting to a diabetes or prediabetes diagnosis (National Institute for Health and Care Excellence guidelines, https://www.nice.org.uk/guidance/NG28).^237^, ^238^ Screening for diabetes in the dental setting was found to be effective in identifying both prediabetes and diabetes, leading to improved glycemic control in a study in the Unites States.^239^ The National Health and Nutrition Examination Study 2013‐2016 study estimated that screening for prediabetes in the dental office might detect around 22.36 million adults with risk of prediabetes or diabetes.^240^ Furthermore, diabetes risk assessment and education by dental professionals of affected not previously diagnosed subjects may contribute to improved patient outcomes, given the effect of uncontrolled diabetes on wound healing.^64^, ^241^ Equally, medical practitioners and diabetologists should be alert to the possible presence of periodontal disease in patients affected by diabetes, bearing in mind that periodontal treatment might help in the management of type 2 diabetes, and the importance of periodontal prevention.^40^ Attention to the patient's medical history is one of the cornerstones for efforts in the prevention of periodontal and peri‐implant diseases.^242^

A joint workshop between the European Federation of Periodontology and the International Diabetes Federation has recently suggested guidelines for the managements of patients with diabetes mellitus.^212^ Based on these guidelines and on the overall evidence discussed in the present paper, the following specific concepts should be borne in mind when dealing with the diabetic patient:

- Specific oral health education should be provided to patients with diabetes mellitus, including discussions about increased risk of periodontitis and about its negative impact on metabolic control and increased risk of diabetes complications.
- Well‐controlled diabetes mellitus does not seem to affect the risk of developing periodontitis, peri‐implantitis, or response to periodontal treatment.
- Poorly controlled diabetes mellitus increases the risk of periodontitis, peri‐implantitis, and poor response to periodontal therapy. Therefore, every effort should be made to prompt patients to a suspected diagnosis of diabetes mellitus (when they are unaware) and to the correct management.
- Periodontal treatment might help in the management of diabetes by helping reduce glycated hemoglobin levels, at least in the short term.
- The need for extensive oral surgery should be assessed with caution with the treating physician and patient in poorly controlled diabetic patients in order to avoid both hypoglycemia and healing complications.
- Supportive periodontal care needs to take into account the elevated risk of periodontal complications in poorly controlled diabetic patients.
- Physicians should investigate the presence of signs or symptoms of periodontal disease as part of a diabetes care visit and, if appropriate, ascertain that periodontal care is being provided. Furthermore, patients should be informed about a higher risk of oral conditions such as dry mouth, burning mouth, and fungal infections.
- Patients with diabetes who have extensive tooth loss should be encouraged to pursue dental rehabilitation to restore adequate mastication for proper nutrition.

## CONFLICTS OF INTEREST

The authors have stated explicitly that they have no conflicts of interest in connection with this article.
